# Fcγ-receptor-activation by circulating immune complexes in systemic autoimmune diseases and its reduction by CD19-CAR T cell therapy

**DOI:** 10.1093/rheumatology/keaf627

**Published:** 2025-12-03

**Authors:** Merle Freitag, Philipp Kolb, Valeria Falcone, Maren Claus, Pauline Schröder, Ivana Andreeva, Lea Rodon, Meike Ewald, Franca Sophie Deicher, Ayla Nadja Stütz, Jörg H W Distler, Norbert Blank, Ricardo Grieshaber-Bouyer, Luis E Muñoz, Hanns-Martin Lorenz, Georg Schett, Wolfgang Merkt

**Affiliations:** Department of Hematology, Oncology and Rheumatology, Internal Medicine V, University Hospital Heidelberg, Heidelberg, Germany; Institute of Virology, University Medical Center, Freiburg, Germany; Faculty of Medicine, Albert-Ludwigs-University, Freiburg, Germany; Institute of Virology, University Medical Center, Freiburg, Germany; Faculty of Medicine, Albert-Ludwigs-University, Freiburg, Germany; Leibniz Research Center for Working Environment and Human Factors at TU Dortmund (IfADo), Dortmund, Germany; Department of Hematology, Oncology and Rheumatology, Internal Medicine V, University Hospital Heidelberg, Heidelberg, Germany; Department of Hematology, Oncology and Rheumatology, Internal Medicine V, University Hospital Heidelberg, Heidelberg, Germany; Department of Hematology, Oncology and Rheumatology, Internal Medicine V, University Hospital Heidelberg, Heidelberg, Germany; Department of Hematology, Oncology and Rheumatology, Internal Medicine V, University Hospital Heidelberg, Heidelberg, Germany; Department of Hematology, Oncology and Rheumatology, Internal Medicine V, University Hospital Heidelberg, Heidelberg, Germany; Department of Rheumatology, University Hospital Düsseldorf, Medical Faculty of Heinrich-Heine University, Düsseldorf, Germany; Hiller Research Center, University Hospital Düsseldorf, Medical Faculty of Heinrich-Heine University, Düsseldorf, Germany; Department of Rheumatology, University Hospital Düsseldorf, Medical Faculty of Heinrich-Heine University, Düsseldorf, Germany; Hiller Research Center, University Hospital Düsseldorf, Medical Faculty of Heinrich-Heine University, Düsseldorf, Germany; Department of Rheumatology, University Hospital Düsseldorf, Medical Faculty of Heinrich-Heine University, Düsseldorf, Germany; Hiller Research Center, University Hospital Düsseldorf, Medical Faculty of Heinrich-Heine University, Düsseldorf, Germany; Department of Hematology, Oncology and Rheumatology, Internal Medicine V, University Hospital Heidelberg, Heidelberg, Germany; Department of Internal Medicine 3, Rheumatology and Clinical Immunology, Friedrich-Alexander-University (FAU) Erlangen-Nürnberg and Universitätsklinikum Erlangen, Erlangen, Germany; Deutsches Zentrum Immuntherapie (DZI), Friedrich-Alexander University (FAU) Erlangen-Nürnberg and Universitätsklinikum Erlangen, Erlangen, Germany; Department of Internal Medicine 3, Rheumatology and Clinical Immunology, Friedrich-Alexander-University (FAU) Erlangen-Nürnberg and Universitätsklinikum Erlangen, Erlangen, Germany; Deutsches Zentrum Immuntherapie (DZI), Friedrich-Alexander University (FAU) Erlangen-Nürnberg and Universitätsklinikum Erlangen, Erlangen, Germany; Department of Hematology, Oncology and Rheumatology, Internal Medicine V, University Hospital Heidelberg, Heidelberg, Germany; Department of Internal Medicine 3, Rheumatology and Clinical Immunology, Friedrich-Alexander-University (FAU) Erlangen-Nürnberg and Universitätsklinikum Erlangen, Erlangen, Germany; Deutsches Zentrum Immuntherapie (DZI), Friedrich-Alexander University (FAU) Erlangen-Nürnberg and Universitätsklinikum Erlangen, Erlangen, Germany; Department of Hematology, Oncology and Rheumatology, Internal Medicine V, University Hospital Heidelberg, Heidelberg, Germany; Department of Rheumatology, University Hospital Düsseldorf, Medical Faculty of Heinrich-Heine University, Düsseldorf, Germany; Hiller Research Center, University Hospital Düsseldorf, Medical Faculty of Heinrich-Heine University, Düsseldorf, Germany

**Keywords:** circulating immune complexes, Fcγ-receptors, systemic sclerosis, primary Sjögren’s syndrome, connective tissue diseases, interstitial lung disease, autoantibodies

## Abstract

**Objectives:**

The role of autoantibody-producing B cells in connective tissue diseases (CTD) has recently been highlighted by the successful treatment with CD19-targeting CAR T cells. Detrimental effects of autoantibodies are linked to the formation of deposited IgG complexes and the activation of immune cells via Fcγ receptors (FcγRs). The role of circulating immune complexes (cICs) as a link between adaptive and innate immunity has remained understudied. Clinical testing of cICs has been hindered by the lack of reliable detection methods. The aim of this study was to determine the potential of IgG-containing cICs to activate FcγRs (their bioactivity) using a new detection method.

**Methods:**

A reporter cell platform was used to assess the presence and bioactivity of cICs in IgG-autoantibody-positive CTD patients (cross-section analysis) and in patients treated with CD19-CAR T cells (longitudinal analysis).

**Results:**

The bioactivity of cICs in the cohort of patients with CTDs was significantly higher compared with healthy controls and patients with IgG-autoantibody-negative systemic inflammatory disease (psoriatic arthritis). Analyses of individual diseases revealed the presence of cICs in the sera of all CTDs, including systemic sclerosis (SSc) and primary Sjögren’s syndrome, although there was significant heterogeneity among individuals. Within SSc, patients positive for anti-topoisomerase-I (Scl70) autoantibodies, diffuse cutaneous and lung involvement had significantly enhanced cIC bioactivity. Finally, the bioactivity of cICs was significantly reduced in CTD patients after CD19-CAR T cell therapy.

**Conclusions:**

Our study reveals the presence of FcγR-engaging cICs in CTDs and demonstrates that the bioactivity of cICs is correlated with clinical phenotypes and treatment outcomes.

Rheumatology key messages

- Circulating immune complexes (cICs) engage and activate FcγRs in autoimmune-mediated connective tissue diseases.
- In systemic sclerosis, cIC bioactivity associated with anti-topoisomerase-I autoantibodies, higher skin scores and interstitial lung disease.
- Deep B cell depletion by CD19-CAR T cells significantly reduced the bioactivity of cICs.

## Introduction

**Figure 1.**
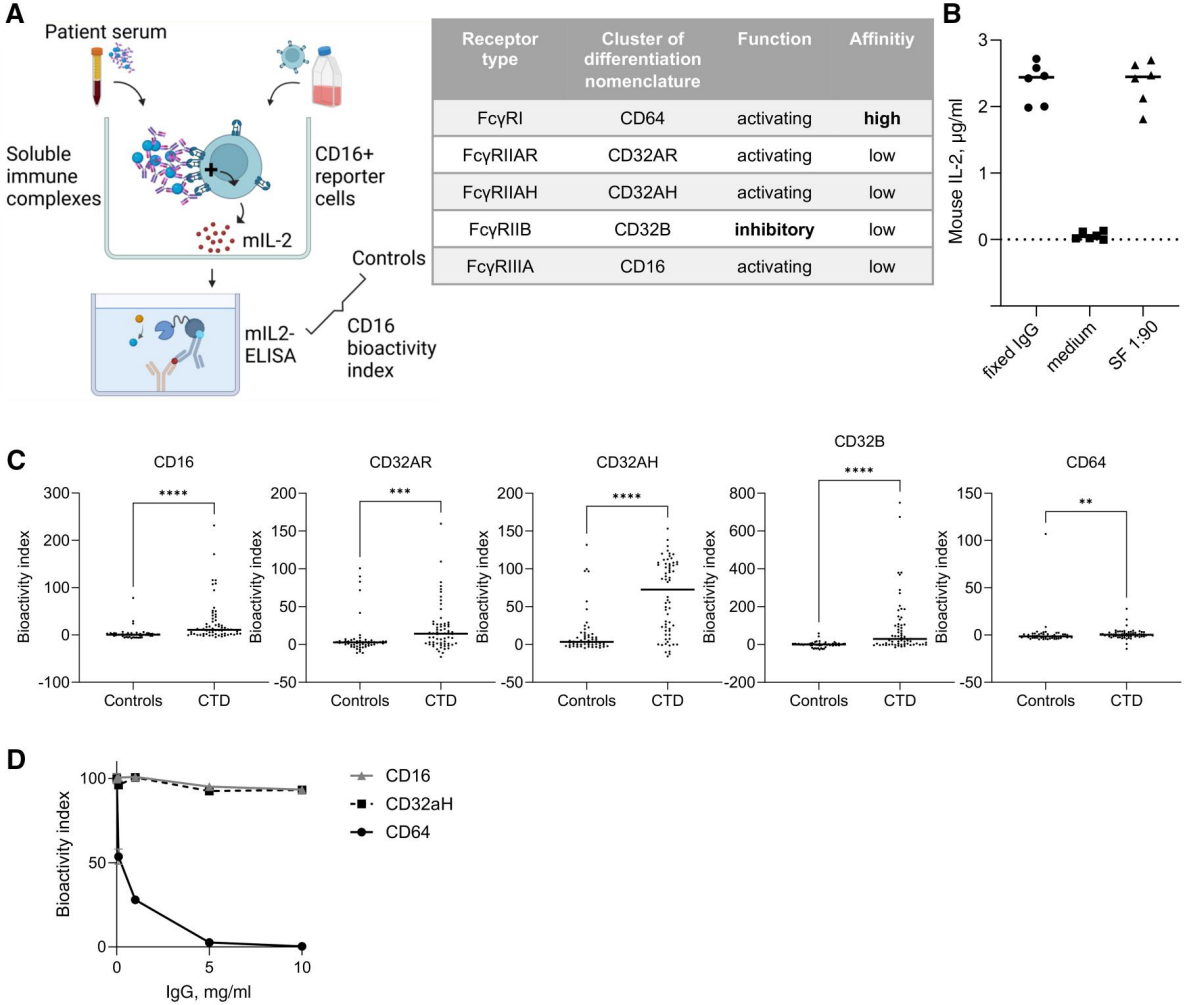
FcγR bioactivity of circulating immune complexes (cICs) in systemic autoimmune diseases. (**A**) Schematic illustration of the reporter cell assay used to determine the bioactivity of cICs. CD16: FcγRIIIA; mIL-2: mouse IL-2. The equation for the calculation of the bioactivity index is provided in the Methods section. (**B**) Positive and negative controls that were used in experiments to control for the functionality of the reporter cells and to calculate the bioactivity indices. As positive controls, immobilized pooled donor IgG from an intravenous immunoglobulin (IvIg) preparation and synovial fluid (SF) with a pre-determined high bioactivity of soluble immune complexes (1:90 dilution) were used, and as negative controls, culture medium without human serum was used. (**C**) Cross-sectional study. A panel of reporter cells was used to determine the capability of sera from connective tissue disease (CTD) patients to activate the Fcγ receptors FcγRIII (CD16), FcγRII (CD32B, CD32AH, CD32AR) and FcγRI (CD64). Controls (*n* = 56) consisted of healthy donors (*n* = 36) and patients with non-IgG-autoantibody-mediated inflammatory disease (psoriasis arthritis; *n* = 20). CTD (*n* = 64) consisted of patients with systemic sclerosis (SSc; *n* = 29), systemic lupus erythematosus (SLE; *n* = 19) and primary Sjögren’s syndrome (SjS; *n* = 16) patients. For statistical analysis, Mann–Whitney tests were applied (not assuming Gaussian distribution, **P < 0.01, ***P < 0.001; ****P < 0.0001). (**D**) Monomeric IgG blocks the engagement of immobilized IgG to FcγRI (CD64) *in vitro*. FcγRI (CD64), FcγRII (CD32AH) and FcγRIII (CD16)-reporter cells were cultured on immobilized IgG. *x*-Axes show monomeric IgG concentration. Symbols represent means of technical triplicates; error bars indicate ranges

In the 1980s, circulating IgG immune complexes (cICs) were proposed to play a central role in the pathophysiology of connective tissue diseases (CTD), such as systemic lupus erythematosus (SLE) and systemic sclerosis (SSc). These complexes of IgG antibodies bound to their respective antigens activate cells through Fcγ receptors (FcγRs), immune receptors that recognize the Fc part of IgG antibodies, which are expressed by most cells of the immune system. Despite growing interest in autoantibodies, the focus on cICs declined due to the low sensitivity and specificity of detection methods. Moreover, there was no method capable of quantifying the bioactivity of cICs, meaning their overall potential to engage and cross-link FcγRs remained unknown. The bioactivity of cICs encompasses all features of complexed IgG antibodies that influence FcγR activation, such as IgG glycosylation, IgG subclass composition, IgG titres and IC size [1–3]. We recently introduced a novel reporter cell panel and assay platform that addressed the limitations in cIC quantification and bioactivity testing. This method enabled us to directly assess the bioactivity of soluble IgG immune complexes in patient samples, including cICs in serum [2, 4, 5] (Fig. 1A). We subsequently reported cICs with high FcγRIIIA(CD16) bioactivity in overshooting immune responses in severe COVID-19, SLE and rheumatoid arthritis (RA) [2, 4, 6]. In RA, soluble ICs possess an especially high bioactivity when derived from the site of inflammation, i.e. in synovial fluid [5, 6]. Beyond these studies, no evidence exists on cIC bioactivity regarding individual FcγRs in systemic autoimmunity. While the presence of bioactive cICs in SLE has been confirmed in two independent cross-sectional studies [6, 7], longitudinal data are lacking in SLE and no data exist in SSc and primary Sjögren’s syndrome (SjS). SSc and SjS are characterized by limited therapeutic options and complex pathophysiology involving fibrotic processes. The exact role of autoantibodies in these conditions remains uncertain. However, it is generally recognized that specific autoantibodies influence the clinical phenotype of SSc and help to define specific subgroups of this disease. An example are anti-topoisomarease-I (Scl70) autoantibodies, which are linked to severe skin and lung involvement in SSc. Therefore, it is important to understand the downstream effects of autoantibodies in CTD, particularly in SSc. Characterization of cICs could further subgroup these diseases, predict outcomes and even identify novel therapeutic targets.

In recent years, deep depletion of B cells by CD19-CAR T cells has been shown to be a highly efficient novel therapeutic strategy for CTD [8–13]. Recent case reports suggested that CD19-CAR T cells also possess great potential in the treatment of SSc [14–18]. In an anti-Scl70-positive SSc patient that developed progressive pulmonary fibrosis despite standard therapies, third-generation CD19-CAR T cell application was associated with a substantial improvement of pulmonary involvement [15, 18, 19]. In this patient, our reporter cell assay detected CD16- and FcγRIIA (CD32A) bioactivity of cICs during clinical progression in the treatment-refractory phase. This case was the first to demonstrate the regression of cIC bioactivity after CD19-CAR T cell therapy in an autoimmune disease [18]. However, confirmation in a larger patient cohort is warranted.

The efficacy of CAR T cells that allowed for off-treatment clinical and serological remissions for over 2 years in certain CTDs [8] indicates that autoantibody-production by the B cell compartment is an early and central component of the complex immunological disturbances in CTDs. The importance of various FcγRs including CD16 and CD32A in CTDs is substantiated by genetic association studies [20]. Thus, in addition to the direct immunological effects of autoantibodies, their downstream effects mediated by the activation of FcγRs likely contribute to the pathophysiology of CTD. While cell/surface-bound immune deposits mediate local inflammation in tissues, cICs may serve as a surrogate and/or active part of early events in CTD pathophysiology, potentially contributing to the systemic spread of the diseases. This hypothesis, however, still requires proof that cICs (i) are activating FcγRs, (ii) correlate with systemic disease manifestations, and (iii) disappear during successful therapies. While individually explored in previous studies, these variables remain to be systematically analysed, particularly in SSc and primary SjS. Notably, cICs can mediate organ damage as shown in murine ILD that is at least partly driven by FcγR activation [21]. Clarifying the bioactivity of cICs is also needed because Fc-receptor blocking strategies emerge in the clinics and possess great potential for the treatment of autoimmunity [22]. Thus, a better understanding of cICs has the potential to pave the way for new treatment strategies in systemic autoimmunity.

In this study, we assessed the bioactivity of cICs using a comprehensive panel of FcγR reporter cells expressing constructs with the extracellular domains of each single FcγR in a cross-sectional study of several different CTDs. In addition, we analysed cIC bioactivity in each of four SSc and SLE patients before and after CD19-CAR T cell therapy.

## Methods

### Patients, clinical data and materials

The local institutional review boards approved this study (University of Heidelberg, S-272/2021). Patients with SSc (*n* = 29), SLE (*n* = 19), primary SjS (*n* = 16), psoriatic arthritis (PSA, *n* = 20) and healthy controls (*n* = 36) were included in the study (Table 1). Written informed consent was obtained from every donor. Serum was frozen and stored until the experiments were carried out. Characteristics of patients are provided in Tables 1 and 2 and the Supplementary Tables. All patients fulfilled current classification criteria [23–26]. Anti-dsDNA antibodies were determined in the routine clinical lab; depending on data availability, either results from ELISA or RIA were used.

**Table 1.** Characteristics of patients and controls included in the cross-sectional study

	SSc (*n* = 29)	SLE (*n* = 19)	Primary SjS (*n* = 16)	PSA (*n* = 20)	Healthy donors (*n* = 36)
Demographic data					
Age, median (range), years	55 (40–81)	28.5 (18–68)	59 (34–79)	58 (32–75)	25 (19–61)
Sex, female, *n* (%)	23 (79)	19 (100)	16 (100)	12 (60)	24 (67)
Duration of disease, median (range), years	10 (0–26)	7 (1–24)	8.5 (1–27)	8.5 (1–41)	
Autoantibodies					
ANA, *n* (%)	29 (100)	19 (100)	15 (94)	4 (20)	
Scl70, *n* (%)	20 (69)				
Anti-centromere-antibodies, *n* (%)	7 (24)				
dsDNA [at donation], *n* (%)		9 (47)			
C3 < 0.9 g/l [at donation], *n* (%)		7 (37)			
≥2 autoantibodies, *n* (%)	5 (17)	18 (90)	14 (88)	2 (10)	
Laboratory tests					
Elevated CRP, *n* (%)	7 (24)	4 (20)	5 (31)	3 (15)	
Selected organ manifestations					
Raynaud, *n* (%)	29 (100)	6 (30)	3 (19)	0 (0)	
Digital ulcers, *n* (%)	20 (69)	7 (35)	0 (0)	0 (0)	
mRSS, mean (range)	10 (0–32)				
ILD, *n* (%)	15 (52)	1 (5)	0 (0)	0 (0)	

ANA: antinuclear antibody; dsDNA: anti-double stranded DNA antibody; ILD: interstitial lung disease; mRSS: modified Rodnan skin score; PSA: psoriatic arthritis; Scl70: anti-topoisomerase 1 antibody; SLE: systemic lupus erythematosus; SjS: Sjögren’s syndrome; SSc: systemic sclerosis.

**Table 2.** Selected characteristics of patients treated with CD19-CAR T cells

Pat ID (ref. to [17])	Diagnosis	CAR T infusion date	Sample acquisition date		Last rituximab infusion
1	SLE	22.03.2021	Pre	10.03.2021	>6 months ago
	SLE		Post	23.01.2023	
6	SLE	09.05.2022	Pre	25.04.2022	>6 months ago
	SLE		Post	03.07.2023	
7	SLE	26.09.2022	Pre	11.08.2022	<6 months ago
	SLE		Post	14.06.2023	
8	SLE	27.02.2023	Pre	07.02.2023	<6 months ago
	SLE		Post	06.07.2023	
12	SSC	01.08.2022	Pre	04.07.2022	<6 months ago
	SSC		Post	27.02.2023	
13	SSC	05.12.2022	Pre	22.11.2022	—
	SSC		Post	10.07.2023	
14	SSC	31.01.2023	Pre	16.01.2023	—
	SSC		Post	17.07.2023	
15	SSC	22.05.2023	Pre	08.05.2023	—
	SSC		Post	21.08.2023	

### Assessment of FcγR bioactivity of circulating immune complexes

To quantify bioactive cICs, an Fcγ receptor (FcγR) reporter cell panel was used as previously described [2, 4, 5]. Briefly, serum (1:320) was incubated with BW5147 mouse thymoma cells stably expressing chimeric human FcγRs IIIA, IIB, IIAH, IIAR and I (CD16, CD32B, CD32AH, CD32AR and CD64, respectively) which consist of the respective receptor ectodomains genetically fused to the transmembrane domain and cytosolic signalling motif of mouse CD3-zeta. Each reporter cell type expressed only one type of FcγR. Upon receptor cross-linking, these reporter cells release murine IL-2, which is quantified via sandwich ELISA [2]. Immobilized pooled monomeric immunoglobulins (pooled donor IgG/intravenous immunoglobulins, IvIg) and medium without human serum served as positive and negative controls, respectively. cIC bioactivity index was calculated by: (Mean sample − Mean negative control)/(Mean positive control − Mean negative control) × 100. The bioactivity index is a measure of both cIC presence and FcR bioactivity.

### CAR T cells

Second-generation CD19-targeting CAR T cells were generated and applied as reported earlier [16].

### Flow cytometry

Reporter cells were washed, incubated with fluorescence-labelled antibodies as indicated and directly analysed on an LSRII (Becton, Dickinson and Company, Franklin Lakes, New Jersey, USA).

### Statistical analysis

The analysis was purely descriptive. To compare two groups, the Mann–Whitney test (unpaired) or the Wilcoxon matched pairs test (paired analyses) was applied (two-sided if not stated otherwise). To compare more than two groups, Kuskal–Wallis and Dunn’s post tests were applied. To compare frequencies, the χ^2^ test was applied (GraphPad Prism versions 8 and 10, GraphPad Software, Boston, MA, USA). For correlation analyses, Spearman’s test was used (Gaussian distribution was not assumed). Outliers were excluded only when indicated using the *ROUT* method [27] (GraphPad Prism version 10). Asterisks indicate level of significance (*P < 0.05, **P < 0.01, ***P < 0.001, ****P < 0.0001). Multivariate analyses were performed using multiple linear regression with cIC bioactivity indices as dependent variables as well as mRSS as continuous variable and autoantibody status and ILD as categorical variables (yes/no) (GraphPad Prism version 10).

## Results

### Identification of pan-FcγR reactive cICs in SSc, SLE and SjS

Adding to our previous studies that utilized an FcγR reporter cell panel to identify cICs, we expanded our studies to include sera from SSc and SjS patients. The reporter assay is detailed in Fig. 1A. Immobilized human IgG (IvIg) and pooled sera or synovial fluid from seropositive RA with known high sIC bioactivity [6] served as positive controls, while plain medium served as negative control (Fig. 1B). Human sera were diluted in reporter cell medium and incubated with reporter cells expressing FcγRI/CD64, FcγRIIAH/CD32AH, FcγRIIAR/CD32AR, FcγRIIB/CD32B and FcγRIIIA/CD16. Sera from 64 patients with autoantibody-associated CTDs (29 SSc, 19 SLE and 16 SjS) were compared with 56 sera from an autoantibody-negative control group consisting of 36 healthy donors and 20 patients with PSA (Table 1).

CTD sera activated all FcγRs significantly more strongly than the autoantibody-negative control group (Fig. 1C). Activation of the only FcγR with high affinity to monomeric IgG, FcγRI (CD64), by CTD sera was marginal but also statistically significant. This marginal activation of CD64 reporter cells by cICs can be explained by monomeric IgG blocking the engagement of complexed IgG by CD64, while monomeric IgG does not compete with engagement of complexed IgG by CD16 and CD32AH (Fig. 1D) [28].

The bioactivity of cICs is subject to pronounced heterogeneity between individual CTD patients (Fig. 1C). Using correlation matrices, we found that patient sera that activated one type of reporter cell also activated the other types (Supplementary Fig. S1). There were no significant correlations between disease duration and FcγR activation, but there was a tendency for the bioactivity of cICs to be higher in younger CTD patients (Supplementary Fig. S1). Further analysis showed no correlations between FcγR activation and ANA antibody titres, complement C3 and IgG levels (Supplementary Fig. S2). CRP levels did also not correlate with FcγR activation (*r* < 0.3).

### cIC bioactivity is associated with disease manifestations

We next evaluated associations between the bioactivity of cICs and clinical parameters within specific CTD. Patient records were retrospectively analysed (Table 1, Supplementary Tables S1–S11). In SLE, associations of cICs and clinical parameters have been published earlier [6, 7], which is why SLE served as a point of reference in this analysis. Of note and different from previous observations, FcγR-activation did not correlate with anti-dsDNA autoantibodies in SLE patients, which may be explained by the fact that there were mainly SLE patients with well-controlled disease and minimal systemic organ involvement included in this cohort. Hence, only 32% of SLE patients showed reduced complement C3 levels (Supplementary Table S6). Alternatively, different testing of anti-dsDNA autoantibodies might have contributed to this discrepancy.

**Figure 2.**
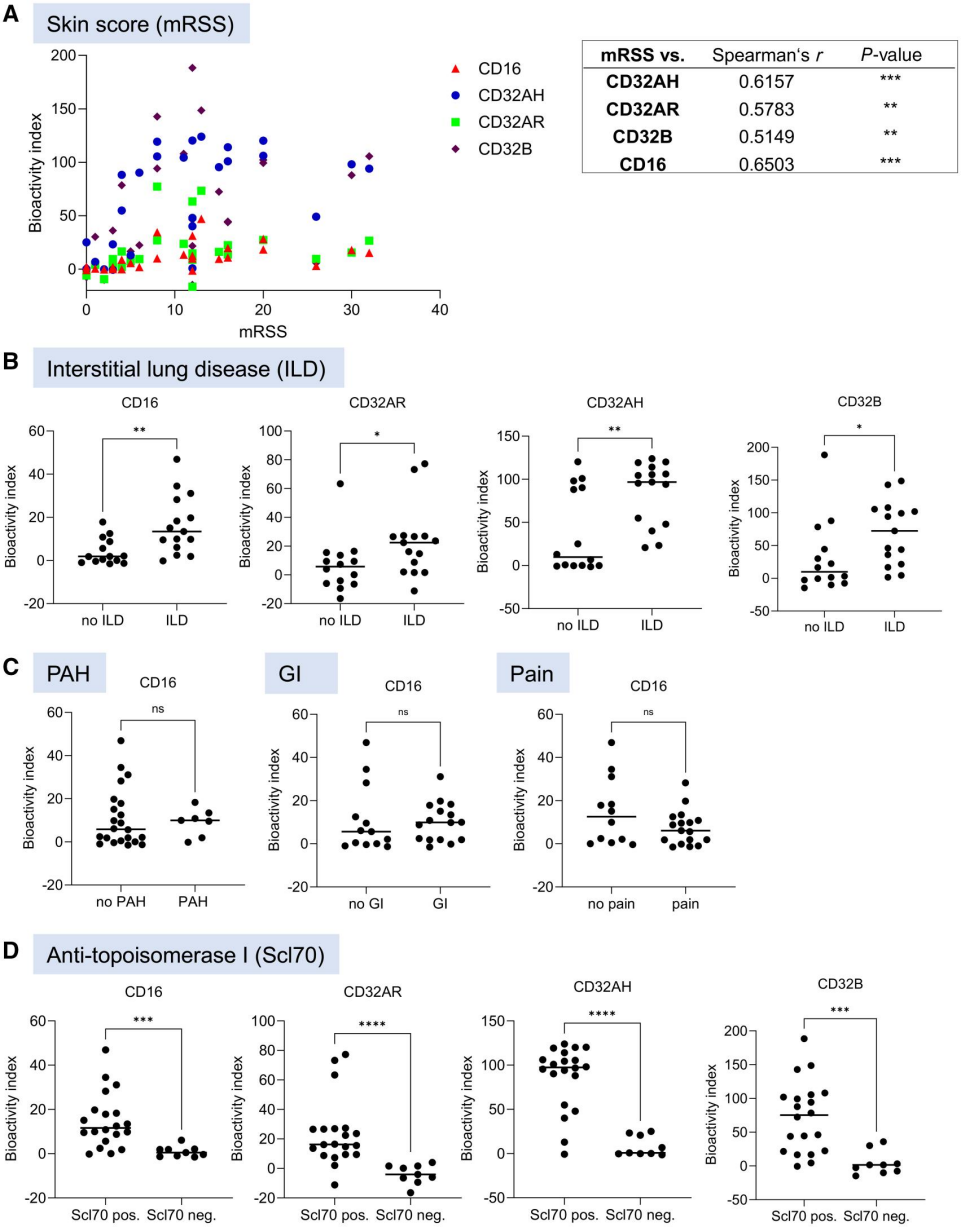
Fcγ receptor activation by circulating immune complexes in systemic sclerosis. (**A**) The modified Rodnan skin score (mRSS) was retrievable from medical records from 26 patients and correlated to the activation of reporter cells containing constructs with the extracellular domains of FcγRI/CD64, FcγRIIAH/CD32AH, FcγRIIAR/CD32AR, FcγRIIB/CD32B and FcγRIIIA/CD16 by serum from these patients using Spearman’s correlation analysis; the table on the right indicates statistical results. (**B–D**) SSc patients were sorted according to organ involvements and autoantibody status (see also Supplementary Tables S1–S4). The activation of FcγRs by SSc sera was plotted depending on absence or presence of (**B**) interstitial lung disease (ILD), (**C**) pulmonary arterial hypertension (PAH), gastrointestinal involvement (GI) and musculoskeletal pain (Pain), and (**D**) autoantibodies against topoisomerase-I (Scl70)

In contrast, the SSc cohort exhibited a significant correlation between the activation of all FcγRs by cICs and the modified Rodnan skin score (mRSS), the standard measure of the extent of skin involvement in SSc. FcγR-activation was significantly higher in SSc patients with interstitial lung disease (ILD) compared with those without ILD (Fig. 2A, B). In contrast, there were no associations between the activation of any of the FcγRs and pulmonary arterial hypertension, gastrointestinal involvement or musculoskeletal pain, as exemplarily shown for CD16 (Fig. 2C). Finally, the activation of all FcγRs was significantly elevated in SSc patients who were positive for autoantibodies against topoisomerase-I (Scl70) compared with those who were not (Fig. 2D). Multivariate analyses confirmed major contributions of the effect sizes of Scl70 status (in particular regarding CD32AH-, CD32AR- and CD32B-activating cICs) and presence of ILD (in particular regarding CD16-activating cICs) but rather low effect sizes of mRSS. These data together indicated that bioactive cICs were especially present in Scl70^+^ SSc with ILD. A subgroup analysis of exclusively anti-Scl70^+^ dcSSc showed a tendency towards more FcγR activation in patients with ILD compared with those without ILD, but patient numbers in the subset without ILD (*n* = 6, 32%) were too low for firm conclusions (Supplementary Fig. S3, Supplementary Table S1).

In SjS, only the activation of CD16 but not that of the other FcγRs showed significant correlation with the serum IgG values (*r* = 0.67, *P < *0.019).

### cICs are found across different CTDs with interindividual heterogeneity

**Figure 3.**
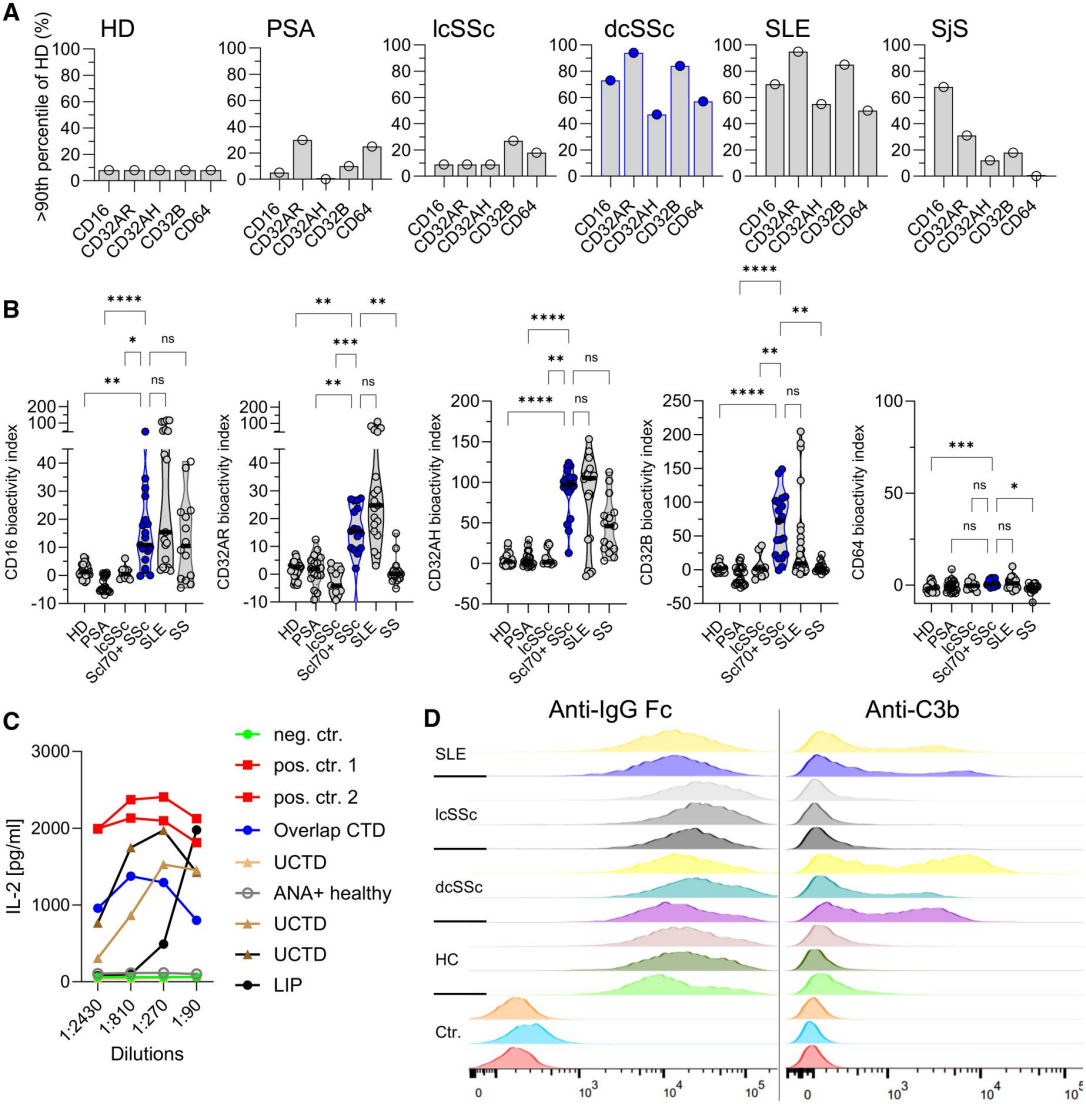
Bioactive cICs are a cross-disease trait in connective tissue diseases (CTDs) despite interindividual heterogeneity. (**A**) Symbols and bars represent the frequencies of sera with FcγR bioactivity above the threshold of normal, which was defined as the 90th percentile of the healthy controls shown on the left; the data were calculated on the basis of the fold over the negative controls. Significant differences in the frequencies of bioactive cICs at each FcγR between patient groups were confirmed by Chi-Square tests (*P* < 0.0001). Systemic sclerosis (SSc) is shown in blue for easier comparison with (**B**). (**B**) Extent of FcγR-activation by cICs between patient groups. The means of experimental doublets are shown after exclusion of outliers using ROUT. For better visualization, only post test results comparing Scl70^+^ dcSSc with the other groups are shown in the graphs. (**C**) Application of the reporter cell model to an additional cohort of patients with undefined CTDs. Shown are serial dilutions of patient sera regarding CD16 activation; concentrations of murine IL-2 secreted by activated FcγRIII (CD16) reporter cells are shown. cICs-containing synovial fluid served as positive control. (For details on patient characteristics see Supplementary Fig. S12.) (**D**) FcγRIII (CD16) reporter cells were probed for human IgG and C3b binding by flow cytometry after incubation with sera from patients (two with SLE, each of three with lcSSc and Scl70^+^ dcSSc) or healthy controls (HC). Shown are histograms of each condition. Ctr.: experimental controls (untreated, autofluorescence, ‘fluorescence-minus one (FMO)’-control); anti-IgG Fc: antibody against human Fc parts; anti-C3b: antibody against human complement factor 3. dcSSc: diffuse cutaneous SSc; HD: healthy donors; lcSSc: local cutaneous SSc; LIP, lymphocytic interstitial pneumonia; PSA: psoriasis arthritis; SLE: systemic lupus erythematosus; SjS: primary Sjögren’s syndrome; UCTD, undifferentiated CTD

Next, we compared the different CTDs and control groups against each other. With the high FcγR activation in Scl70^+^ dcSSc, the SSc cohort was divided into dcSSc (*n* = 19) and lcSSc (*n* = 10) patients (Fig. 3A, Supplementary Tables S1–S4). All patients with dcSSc were positive for anti-topoisomerase-1 (Scl70) autoantibodies, which may be explained by sample collection criteria in our serum bank. In contrast, 70% of lcSSc patients were positive for anti-centromere autoantibodies. The presence of ILD also matched the typical distribution between Scl70^+^ dcSSc and anti-Centromere^+^ lcSSc (65% *vs* 20%).

To compare individual disease entities, the upper range of normal cICs bioactivity was calculated. As low levels of cICs are also found in a subset of healthy subjects [6], the upper range of normal was defined as the 90th percentile of cIC bioactivity levels observed in the healthy control cohort. The percentage of cIC bioactivity-positive patients was particularly high in anti-Scl70^+^ dcSSc patients and in SLE patients (Fig. 3A), which was consistent among all investigated FcγRs. Primary SjS also showed an increased percentage of patients with bioactive cICs, particularly with respect to CD16, whereas PSA and lcSSC showed no increased proportion of patients with FcγR activation by serum.

Next, we compared the extent of FcγR activation by cICs between patient groups (Fig. 3B). Similarly to the analysis of the percentage of cIC-positive individuals, the extent of FcγR-activation by cICs was elevated in CTDs except for lcSSc. However, despite statistical exclusion of outliers using the ROUT method [27], we observed an important heterogeneity within patient groups, especially in SLE and SSc. These data together suggest that cICs are present across all CTDs, yet with heterogeneous distribution and with the exception of lcSSc.

In addition, we also confirmed the presence of FcγR-activating cICs in five patients with CTD that were not falling into the SLE, SSc or SjS classification, including three patients with undifferentiated CTD (UCTD), one overlap-CTD (SjS, myositis and autoimmune hepatitis) and one ANA-positive patient with lymphocytic ILD (Fig. 3C, Supplementary Table S12). In contrast, no FcγR-activating cICs were found in an individual with asymptomatic ANA elevation.

As an additional confirmation of the presence of cICs in Scl70^+^ dcSSc and their absence in lcSSc, we performed a flow cytometry analysis using fluorochrome-labelled anti-human-IgG-Fc and anti-complement factor-C3 antibodies. While CD16-expressing reporter cells were loaded by human IgG-Fc after incubation with human serum from all entities (SLE, lcSSc, Scl70^+^ dcSSc and healthy serum), loading with complement C3 was exclusively found after cultivation with SLE and Scl70^+^ dcSSc sera, but not in lcSSc or healthy sera (Fig. 3D). The presence of C3 confirms the IgG-Fc-complex formation in cICs in sera with bioactivity in our reporter assay.

In summary, cICs were present in a marked proportion of patients with CTD. In patients with SSc, the bioactivity of cICs was heterogeneous and appeared to be restricted to the anti-Scl70^+^ diffuse cutaneous form.

### Longitudinal assessment of cICs during CD19-CAR T cell therapy

**Figure 4.**
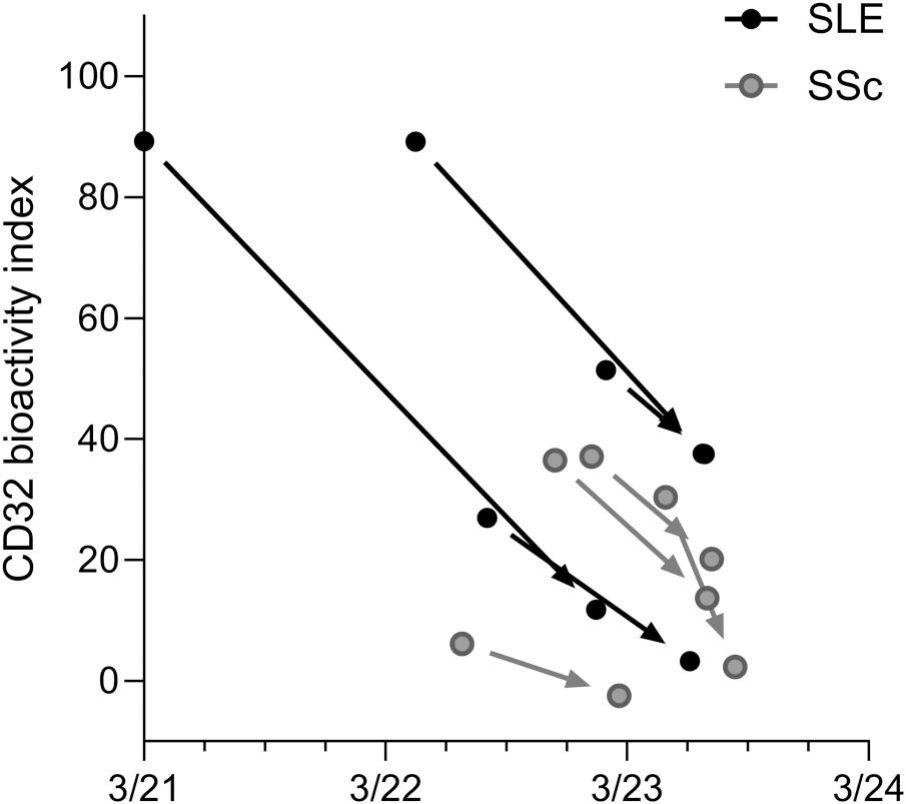
Longitudinal assessment of cICs during CD19-CAR T cell therapy. FcγRII (CD32AH) reporter cells were used to assess bioactivity indices of sera obtained at baseline before the infusion of second-generation CD19-CAR T cells and at follow up. The *x*-axis represents actual dates; each pair of dots linked by an arrow correspond to the same patient with the left dot corresponding to baseline and the right dot to follow-up. SLE and SSc patients are depicted by black and grey dots and arrows, respectively. Of note, dots are partly overlapping. Wilcoxon matched pairs test was significant (*P* = 0.0078)

We measured cICs before and after CD19-CAR T cell therapy in eight patients (four SLE, four SSc). Details about clinical and immunological responses have been reported elsewhere [8, 16]. Time of CAR T cell infusion, time of baseline and follow-up sera acquisitions as well as the interval to the last pre-treatment with rituximab are shown in Table 2. We tested CD32AH reporter cells as these proved to be the most cIC-sensitive reporter cells in our hands, consistent with their original description [7]. CD32AH-activating cICs significantly decreased (*P* = 0.0078) from baseline to follow-up (Fig. 4). Together, CD19-CAR T cells reduced the potency of cICs in patient serum to activate FcγRs.

## Discussion

This study marks the first systematic application of a novel reporter cell model in systemic autoimmune diseases beyond SLE and RA. The study reproduces earlier findings in SLE [6, 7] and establishes the presence of bioactive cICs in SSc and in SjS, suggesting their presence as a general feature in CTD. However, cIC bioactivity was highly heterogeneous between individuals. Causes for this heterogeneity are not fully understood, but may include IgG-related parameters such as glycosylation and subclass composition or the size of cICs. It is striking that a high FcγR-activation by cICs was measured in a significant percentage of SLE patients despite low disease activity and despite missing correlations with clinically established parameters.

Of note, the reporter cell model does not allow for a direct comparison between different Fc receptors, because the level of receptor expression is not normalized. Nevertheless, we observed significant correlations between the activation of activating (CD16, CD32AH, CD32AR) and inhibitory (CD32B) FcγRs, indicating a general capacity of cICs to activate all types of FcγRs. The effect of single nucleotide polymorphisms or the combination of more than one FcγR on single cells was not investigated in this study.

In SSc, the level of relevance of autoimmunity and autoantibodies in the pathophysiology of the disease is still debated. Several lines of evidence support a causative role of adaptive immunity in SSc, including single nucleotide polymorphisms in immune-regulating genes [29, 30], a disturbed B cell homeostasis [31] and a partial response to B cell depletion [17, 18, 32–38]. Additionally, an important role of autoimmunity can be deduced by clinical associations between distinct autoantibody types and SSc-phenotypes, with anti-Scl70 autoantibodies characterizing a systemic inflammatory form of the disease including ILD [39, 40].

The bioactivity of cICs, including cross-linking of CD16, was significantly elevated in sera from anti-Scl70^+^ SSc patients that suffered from severe skin fibrosis and ILD, but not in patients with lcSSc. Interestingly, polymorphisms in the CD16 gene are associated with the emergence of Scl70 autoantibodies and ILD in SSc [41], and the common γ-chain signalling module of CD16, CD247, is a known risk locus for SSc [30]. Previous studies, based on indirect evidence, also suggested that cICs were more commonly found in Scl70^+^ dcSSc than in anti-Scl70-negative SSc [42–44]. Together with these studies, our findings suggest a role of cIC-induced FcγR-signalling, particularly of CD16, in the pathogenesis of Scl70^+^ SSc. In the light of preclinical evidence showing that cICs can induce lung injury by FcγR-activation [21] and the clinical associations described in our study, it is tempting to speculate that bioactive cICs in Scl70^+^ SSc contribute to the development of ILD. Hypothetically, the association between anti-Scl70 autoantibodies and cICs could imply that the formation of cICs depends on the presence of Scl70-antigen. In support of this idea, we have recently demonstrated that adding Scl70-antigen to serum from anti-Scl70^+^ SSc patients resulted in the formation of soluble immune complexes capable of activating CD16-bearing NK cells *in vitro* [36]. This observation is corroborated by *in vivo* data showing that the activated NK cell phenotype in an Scl70^+^ SSc patient was abrogated after CD19-CAR T cell treatment in parallel to the disappearance of cICs and anti-Scl70 autoantibodies as well as regression of ILD [15, 18, 19, 36]. Data in this paper show that CD19-CAR T cell treatment significantly decreased bioactive cICs in both SLE and SSc patients.

Compared with SSc, SjS is characterized by less disease-specific autoantibodies and by highly variable degrees of systemic inflammation. We found CD16-activating cICs in SjS, while data on CD32AH, CD32AR, CD32B and CD64 were less conclusive. Whether this means that cICs in SjS differ from SLE and SSc in terms of cIC size or rather composition or abundance needs to be clarified in future studies.

In summary, this study provides evidence that (i) bioactive cICs are present in SSc and SjS patients, (ii) cIC bioactivity associates with disease manifestations in SSc, and (iii) cIC bioactivity regresses upon treatment with CD19-CAR T cells. Data of this study provide new insights into the role of cICs in CTDs, supporting the hypothesis that cICs may be part of CTD pathophysiology. This strongly emphasizes the need for further research on the presence of functional cICs in CTD and the role of immune interventions on cICs.

## Statement on use of artificial intelligence tools

During the preparation of this work the authors used Deepl.com and GPT-4o in order to improve the readability and language of the manuscript. The authors carefully reviewed and edited the content as needed and take full responsibility for the content of the published article.

## Supplementary Material

keaf627_Supplementary_Data

## Data Availability

All relevant data are shown in the manuscript and supplementary materials. Additional data on patient records, procedural details or performed supplementary experiments can be obtained from the corresponding author upon reasonable request.
