# Supplementary material for: Fcγ-receptor-activation by circulating immune complexes in systemic autoimmune diseases and its reduction by CD19-CAR T cell therapy
Source: Rheumatology (Oxford). 2025 Dec 3;65(3):keaf627. doi: 10.1093/rheumatology/keaf627 (PMC13017110; doi:10.1093/rheumatology/keaf627)
Supplement: keaf627_Supplementary_Data [file keaf627_supplementary_data.zip › rhe-25-1615-File005.pdf]

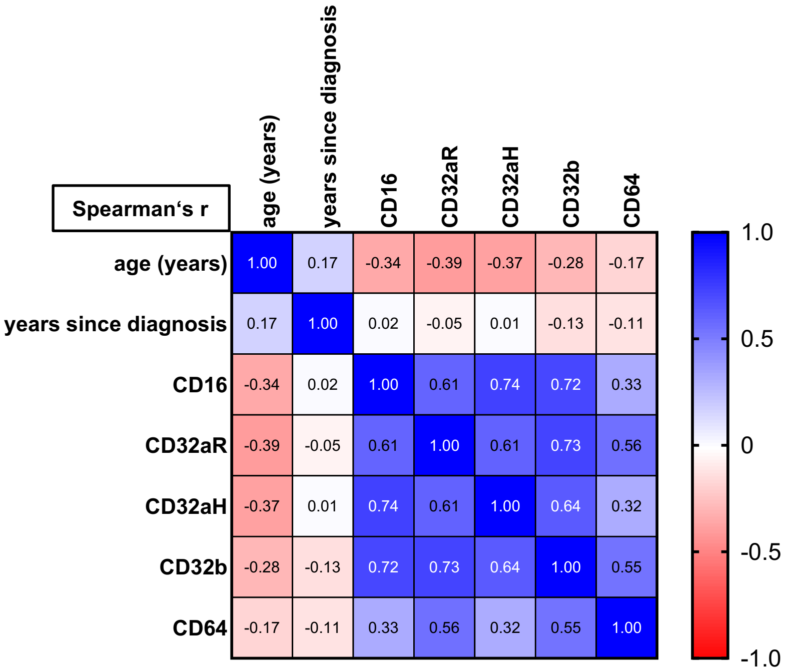

Approximate p values	age (years)	years since diagnosis	CD16	CD32aR	CD32aH	CD32b	CD64
**age (years)**		0,2355	0,0015	0,0000	0,0009	0,0055	0,0302
**years since diagnosis**	0,2355		0,7465	0,2119	0,6639	0,1000	0,1924
**CD16**	0,0015	0,7465		0,0000	0,0000	0,0000	0,0021
**CD32aR**	0,0000	0,2119	0,0000		0,0000	0,0000	0,0000
**CD32aH**	0,0009	0,6639	0,0000	0,0000		0,0000	0,0002
**CD32b**	0,0055	0,1000	0,0000	0,0000	0,0000		0,0000
**CD64**	0,0302	0,1924	0,0021	0,0000	0,0002	0,0000	

Sample size	age (years)	years since diagnosis	CD16	CD32aR	CD32aH	CD32b	CD64
**age (years)**	64	64	63	63	63	63	63
**years since diagnosis**	64	64	63	63	63	63	63
**CD16**	63	63	63	63	63	63	63
**CD32aR**	63	63	63	63	63	63	63
**CD32aH**	63	63	63	63	63	63	63
**CD32b**	63	63	63	63	63	63	63
**CD64**	63	63	63	63	63	63	63

Suppl. Fig. 1

Spearman‘s r	IgG	C3 [g/l]	ANA	CD16	CD32aR	CD32aH	CD32b	CD64
IgG	1.00	-0.13	0.49	0.47	0.17	0.26	0.22	0.04
C3 [g/l]	-0.13	1.00	-0.20	-0.16	-0.34	-0.29	-0.05	-0.25
ANA	0.49	-0.20	1.00	0.40	0.14	0.35	0.22	0.15
CD16	0.47	-0.16	0.40	1.00	0.70	0.72	0.60	0.38
CD32aR	0.17	-0.34	0.14	0.70	1.00	0.69	0.86	0.64
CD32aH	0.26	-0.29	0.35	0.72	0.69	1.00	0.59	0.46
CD32b	0.22	-0.05	0.22	0.60	0.86	0.59	1.00	0.68
CD64	0.04	-0.25	0.15	0.38	0.64	0.46	0.68	1.00

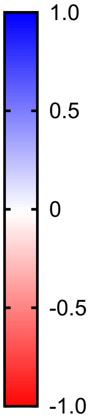

Approximate p value	IgG	C3 [g/l]	ANA	CD16	CD32aR	CD32aH	CD32b	CD64
**IgG**		0,6984	0,0150	0,0227	0,4421	0,2255	0,3223	0,8702
**C3 [g/l]**	0,6984		0,3278	0,4340	0,0783	0,1436	0,7922	0,2138
**ANA**	0,0150	0,3278		0,0013	0,2850	0,0053	0,0876	0,2500
**CD16**	0,0227	0,4340	0,0013		0,0000	0,0000	0,0000	0,0021
**CD32aR**	0,4421	0,0783	0,2850	0,0000		0,0000	0,0000	0,0000
**CD32aH**	0,2255	0,1436	0,0053	0,0000	0,0000		0,0000	0,0002
**CD32b**	0,3223	0,7922	0,0876	0,0000	0,0000	0,0000		0,0000
**CD64**	0,8702	0,2138	0,2500	0,0021	0,0000	0,0002	0,0000	

Sample size	IgG	C3 [g/l]	ANA	CD16	CD32aR	CD32aH	CD32b	CD64
**IgG**	24	11	24	23	23	23	23	23
**C3 [g/l]**	11	27	27	27	27	27	27	27
**ANA**	24	27	64	63	63	63	63	63
**CD16**	23	27	63	63	63	63	63	63
**CD32aR**	23	27	63	63	63	63	63	63
**CD32aH**	23	27	63	63	63	63	63	63
**CD32b**	23	27	63	63	63	63	63	63
**CD64**	23	27	63	63	63	63	63	63

Suppl. Fig. 2

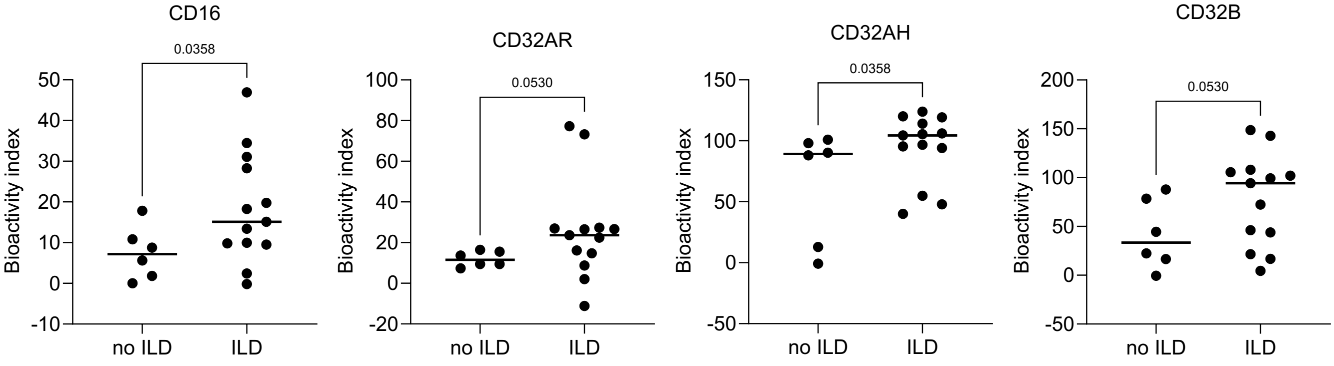
CD16
0.0358
Bioactivity index
50
40
30
20
10
0
-10
no ILD
ILD
CD32AR
0.0530
Bioactivity index
100
80
60
40
20
0
-20
no ILD
ILD
CD32AH
0.0358
Bioactivity index
150
100
50
0
-50
no ILD
ILD
CD32B
0.0530
Bioactivity index
200
150
100
50
0
-50
no ILD
ILD

Suppl. Fig.3
