# Supplementary material for: Fcγ-receptor-activation by circulating immune complexes in systemic autoimmune diseases and its reduction by CD19-CAR T cell therapy
Source: Rheumatology (Oxford). 2025 Dec 3;65(3):keaf627. doi: 10.1093/rheumatology/keaf627 (PMC13017110; doi:10.1093/rheumatology/keaf627)
Supplement: keaf627_Supplementary_Data [file keaf627_supplementary_data.zip › rhe-25-1615-File004.docx]

## Supplementary figures legends

**Supplementary Figure S1. Correlation analyses including disease duration, age and the activation of various Fcɣ receptor-bearing reporter cells by circulating immune complexes.** Sera from patient with autoantibody-positive connective tissue diseases (CTD) were diluted in assay medium and incubated with reporter cells containing constructs with the extracellular domains of FcγRI/CD64, FcγRIIAH/CD32AH, FcγRIIAR/CD32AR, FcγRIIB/CD32B and FcγRIIIA/CD16. The correlation matrix was caluclated using Graphpad's prism version 10. The color code of the heatmap represents spearman's r, the tables below the corresponding p values the sample size of each correlation analysis.

**Supplementary Figure S2. Correlation analyses including anti-nuclear antibody (ANA) titers, complement C3; IgG levels and the activation of various Fcɣ receptor-bearing reporter cells by circulating immune complexes.** Sera from patient with autoantibody-positive connective tissue diseases (CTD) were diluted in assay medium and incubated with reporter cells containing constructs with the extracellular domains of FcγRI/CD64, FcγRIIAH/CD32AH, FcγRIIAR/CD32AR, FcγRIIB/CD32B and FcγRIIIA/CD16. The correlation matrix was caluclated using Graphpad's prism version 10. The color code of the heatmap represents spearman's r, the tables below the corresponding p values the sample size of each correlation analysis.

**Supplementary Figure S3. Fcɣ receptor activation by circulating immune complexes in anti-Scl70+ systemic sclerosis depending on presence of interstitial lung disease (ILD).** Sera from anti-Scl70 autoantibody positive systemic sclerosis patients without (n=6) and with (n=13) ILD were incubated with reporter cells containing constructs with the extracellular domains of FcγRIIAH/CD32AH, FcγRIIAR/CD32AR, FcγRIIB/CD32B and FcγRIIIA/CD16. Exact p values resulting from Mann-Whitney tests (one-sided) are shown in each graph.
